# Atlanta Medical College Commencement

**Published:** 1859-10

**Authors:** 


### EDITORIAL AND MISCELLANEOUS.

**ATLANTA MEDICAL COLLEGE—COMMENCEMENT.**

On the first Thursday of the present month closed the fifth regular course of instruction in the aforesaid Institution, and the large concourse of citizens assembled to witness the annual commencement celebration, bore ample testimony to the interest, on part of this enlightened community, in our flourishing Institution, and the youth committed to our charge. If the Atlanta Medical College required any further evidence of its continued prosperity and brightening future, the exercises of the present day teem with abundant and overwhelming testimony. Our opinions of the class, as a whole, have been fully set forth in a previous number of this journal. But for the graduatihg class, we must say that it has never fallen to our lot to witness the conference of degrees upon a body of young men giving more satisfactory evidence of their fitness, worthiness to receive them—either in intelectual developement, moral status or professional acquirement than these.

The present day brings further indubitable evidence of the steady and rapid increase numerically in our classes—of the improvement in mental qualification, previous educational advantages, and moral fitness to assume those responsibilities now soon to be imposed.

The degree of Doctor of Medicine was conferred on fifty-six young gentlemen, and two graduates of other Institutions were admitted "*Ad Eundem Gradum.*" The diplomas were presented by Prof. Alexander Means, M. D., whose long experience as a teacher has served to render more exquisite his appreciation of the wants, and to increase the deep interest he has ever manifested in the youth of our own Southern clime. His brief, but appropriate and impressive charge fell upon good soil. May it spring up in future years to bear fruit whose flavor and beauty shall be known to time and recognised as immortal.

The Address to the Graduating Class on part of the Faculty, delivered by Eben Hillyer, M. D., of Rome, Ga., chaste, elegant, and impressive, abounded in wholesome advice to the young physician; warning him of the rock of empiricism on the one hand, upon which so many hopes are wrecked, and of the whirlpool of intemperance and sensuality on the other, engulphing too often the brightest and best of our land, as pearls 'neath an oblivious wave to rest. The address we hope to have for publication.

We cannot fail to add our high appreciation of the valedictory delivered by Mr. B. F. Ward, of Miss., one of the graduating class. Rich in thought, and abounding in deep pathos, it bore ample testimony to the high mental culture of the speaker, as the development of the heart's best feelings, which will insure to him the confidence, esteem and respect of all who know him. While we give to each and all our hearty God-speed, we cannot forbear expressing our confidence in those who now have committed to them, in part, the honor and character of their "Alma Mater," and chronicling this day as one, if not the brightest, the most pregnant of promise, that has blessed our young and thriving institution.

